# Authors' Reply

**DOI:** 10.1371/journal.pmed.0030245

**Published:** 2006-05-30

**Authors:** David Wendler, Ezekiel J Emanuel

**Affiliations:** **1**National Institutes of HealthBethesda, MarylandUnited States of America

The letter by Matthew Wynia and Vanessa Northington Gamble is based on an unfortunate conflation of behavior and attitudes [ 1]. Our paper evaluated the often repeated claim that individuals from minority groups are less willing to participate in health research compared with non-Hispanic whites [ 2]. That is, we evaluated what individuals do, whether they give consent, when invited to participate in health research. We did not and did not claim to evaluate individuals' attitudes toward these requests or toward those who make them.

Our findings, based on the existing empirical data, suggest that individuals from minority groups who are eligible and who are invited to participate agree to enroll in health research at rates similar to those of non-Hispanic whites. Wynia and Northington Gamble read this conclusion as a claim about individuals' attitudes, as a denial of the “substantial body of research that demonstrates how common mistrust of the health-care system is among African Americans.” This conflation of individuals' behavior related to research enrollment with their levels of trust in the health-care system is surprising given that our focus on individuals' willingness to participate in research is described in the title, and described throughout the text. Indeed, in an attempt to avoid just this conflation, we explicitly wrote in the text that “we did not assess minority groups' attitudes toward health research.”

Wynia and Northington Gamble also accuse us of ignoring the heterogeneity in the empirical data we found, and claim that “very little information can be reliably gleaned from pooling” these data. With this latter statement, we agree. As explained in the manuscript, the heterogeneity of the data suggests there is no simple relationship between one's race or ethnicity and one's willingness to participate in health research. While little else can be gleaned from these data, their very heterogeneity undermines the claim that individuals from minority groups are consistently less willing than non-Hispanic whites to participate in health research.

## References

[pmed-0030245-b1] Wynia MK, Gamble VN (2006). Mistrust among minorities and the trustworthiness of medicine. PLoS Med.

[pmed-0030245-b2] Wendler D, Kington R, Madans J, Wye GV, Christ-Schmidt H (2006). Are racial and ethnic minorities less willing to participate in health research?. PLoS Med.

